# Non-Uniform Clustering Algorithm for UWSNs Based on Energy Equalization

**DOI:** 10.3390/s23125466

**Published:** 2023-06-09

**Authors:** Jinwang Yi, Jie Tang, Fei Yuan, Guanhao Qiao, Dongping Dai

**Affiliations:** 1 Fujian Key Laboratory of Communication Network and Information Processing, Xiamen University of Technology, Xiamen 361024, China; jwyi@xmut.edu.cn (J.Y.); 2022031160@s.xmut.edu.cn (G.Q.); 2122031244@s.xmut.edu.cn (D.D.); 2 Key Laboratory of Underwater Acoustic Communication and Marine Information Technology, Xiamen University, Xiamen 361005, China; yuanfei@xmu.edu.cn

**Keywords:** energy equalization, non-uniform cluster, underwater sensor networks

## Abstract

Underwater sensor nodes are usually deployed by ships, aircraft, etc., in random drops, and there is current movement in the underwater environment, which results in an uneven distribution of sensor nodes and thus, different energy consumption in each area of the network. In addition, the underwater sensor network also has a “hot zone” problem. To address the uneven energy consumption of the network caused by the above problem, the non-uniform clustering algorithm for energy equalization is put forward. Considering the residual energy, density and coverage redundancy of nodes, this algorithm selects the cluster heads and makes them more reasonably distributed. Additionally, according to the selected cluster heads, the size of each cluster is designed to equalize the energy consumption of the network during multi-hop routing. In this process, the residual energy of cluster heads and the mobility of nodes are considered, and real-time maintenance is performed for each cluster. The simulation results demonstrate that the proposed algorithm is effective in prolonging the network lifetime and balancing the network energy consumption; moreover, the network coverage maintenance is better than that of other algorithms.

## 1. Introduction

With the invention of wearable devices and some of their hardware devices, and the continuous research on efficient communication protocols, underwater wireless sensor networks (UWSNs) have attracted more attention from researchers and made great progress in recent years [1]. Research on UWSNs is divided into many directions, such as node deployment, node localization, and routing protocols, among which the most complex and important is the study of routing protocols [2]. This is because UWSNs are subject to a variety of challenges and limitations: limited battery energy of sensor nodes, dynamic network topology, narrow bandwidth, high propagation delay, high noise, and high ocean interference. The design of routing protocols is one of the solutions to these problems, which can efficiently transmit data from source nodes to destination nodes in the network [3].

Usually, hardware devices such as underwater wearable devices, drifters, hydroacoustic modems [4], and senor nodes are low-power devices. Moreover, UWSNs, unlike terrestrial sensor nodes, are powered by batteries and cannot collect solar energy. Due to the harsh underwater environment, it is very burdensome to recover the batteries [5]. Therefore, to make the sensor network work longer, balancing the network energy consumption and extending the network lifetime are the key goals of routing protocols. In addition, the influence of dead nodes on the monitoring area should be also considered in the routing process to ensure the reliability of the network. Extensive work has shown that cluster routing protocols and multi-hop routing are effective in finding the optimal routing path for data transmission, saving energy, and extending the network lifetime [6].

However, multi-hop routing suffers from the hot zone problem because sensor nodes near the receiver can quickly exhaust their energy and these nodes can die earlier, thus leading to the inability to observe the area of interest [7]. At the initial stage of node deployment for the underwater sensor network, the sensor nodes are often randomly distributed on the water surface by aircraft or ship [8]. The presence of underwater current motion can lead to the uneven distribution of underwater sensor nodes [9], and thus the selection of cluster heads for the clustering algorithm and cluster size is key to avoiding excessive load differences between clusters in sparse and dense areas of nodes. At the same time, in UWSNs based on cluster architecture, when cluster heads transmit data to sink nodes with single-hop or multi-hop communication, they run out of energy and die prematurely due to large amounts of data overload, leading to early blindness in coverage of the monitored area [10]. In addition, due to ocean current activities and other natural factors, underwater nodes are prone to drift, causing the dynamic topological evolution of the network [11]. Therefore, the underwater routing protocol should be scalable to accommodate the dynamic topological change and stability in the network for different emergencies [3].

To address the above problems, an energy-balanced non-uniform clustering (NUC-EB) algorithm is proposed for the underwater sensor network in this paper. The clustering-based multi-hop transmission mechanism is used to balance the network energy consumption and extend the network lifetime. The algorithm first considers the number of neighbor nodes and the distance to each neighbor node and constructs a node density weight function to make cluster heads more evenly distributed in the network. Then, the coverage redundancy index is introduced by cluster heads to improve the fault tolerance of the detection area for cluster heads. Finally, the cluster maintenance mechanism is designed by considering the water flow and the residual energy of cluster heads.

The remainder of this paper is organized as follows: Section 2 reviews the related work on the routing protocols of UWSNs; Section 3 presents the energy consumption model and the underwater sensor network model; Section 4 describes the current algorithm flow; Section 5 evaluates the network performance compared to LEACH, UCUBG and DBR routing protocols; and Section 6 concludes this paper.

## 2. Related Works

UWSNs have been under research over the last decade and researchers have proposed different routing algorithms to extend the network lifetime and reduce high energy consumption.

In [12], a classical depth-based routing (DBR) protocol, also known as a locality-free protocol, is proposed. This algorithm uses a greedy flooding strategy, in which sensor nodes calculate the depth using depth sensors, and high-depth sensor nodes transmit data packets to low-depth sensor nodes until they reach the sink. The biggest advantage of this algorithm is that data can be transmitted to the sink based on the simple routing principle. However, this protocol also has certain drawbacks. First, data may be sent in different ways, possibly leading to high energy consumption. The protocol has a low data transfer rate in sparse networks. Second, due to convergence behavior, failures occur earlier in sensor nodes close to the sink.

In [13], the proposed EEDBR is an extension of the DBR protocol, which selects the optimal forwarding node while combining the residual energy parameter and depth. The sensor node checks the depth and residual energy of the receiving node with itself. Among the neighboring nodes, the node with the smallest depth value and the largest residual energy becomes the next hop destination. Although this algorithm equalizes the network energy consumption to some extent, there is still a poor energy-saving effect.

All of the above are planar routing protocols. The principle of planar routing protocols is usually simple, and the energy consumption problem of the network can be solved to some extent when considering the energy factor. However, cluster routing protocols have more advantages than planar routing protocols [14,15]. Firstly, cluster routing protocols consume less energy and have an evenly distributed energy consumption, which is effective in prolonging the network lifetime and balancing the network load. Secondly, cluster routing protocols are based on some cluster formation strategies that choose to produce a more stable subnetwork, thus reducing the impact of topological changes on the routing protocols. Moreover, cluster head nodes manage nodes within the cluster and can easily communicate various information about the nodes to the base station, such as energy, security, failure, etc. In addition, the base station can effectively send commands to other nodes in the network through the head node, which cannot be effectively achieved by flat routing.

In [16], the adaptive low-power LEACH algorithm is proposed, which is a classical clustering algorithm that uses a wheel-based sensor network for topological reconstruction and uses a probability function to choose the cluster head. This algorithm uses a single-hop transmission mechanism and fails to consider the uneven distribution of cluster heads and the different sizes of each cluster, which can easily lead to the premature death of cluster heads due to uneven energy consumption.

In [17], the authors improved the LEACH algorithm by proposing the DEEC algorithm. This algorithm introduces the residual energy of nodes in the cluster head selection process to increase the probability of nodes with higher residual energy to become cluster heads. It can further extend the network life cycle but does not consider the impact of node density on the network life cycle. The cluster heads are also used for single-hop transmission with poor scalability, which is not applicable to large wireless sensor networks.

In [18], a multi-hop non-uniform clustering algorithm (EEMUC) is implemented in UWSNs. Based on the distance between the node and the sink, the whole network can be divided into an uneven arc-shaped layered model centered on the sink. The nodes in each layer select cluster heads according to the integrated attribute values and reduce the clusters close to the sink. The multi-hop data transmission is used between the clusters to effectively equalize the energy consumption of cluster heads. However, the effect of uneven node distribution on the selection of cluster heads is not considered.

In [19], the proposed clustering algorithm (EGRCs) for data transmission classifies the whole 3D network into multiple grids, with each grid forming a cluster. The node with the largest residual energy and the shortest distance to the sink is selected as the cluster head. The next hop cluster head node is chosen according to the residual energy, end-to-end delay, and node-to-sink distance. This algorithm can prolong the network lifetime and improve the efficiency of energy usage, but it also fails to consider the impact of uneven node distribution on the network survival time based on grid clustering.

In [20], another clustering protocol scheme is applied to multi-hop routing for UWSNs, which selects cluster heads by considering the node coverage redundancy. It is a metric that characterizes the extent to which the sensory area of a node is covered by neighboring nodes. The greater the coverage redundancy, the smaller the impact on the network coverage upon the death of a node. The distributed network unevenly layered coverage preserving routing algorithm (NULCPR) effectively reduces the impact of cluster head mortality on network coverage for each cluster.

In [21], an energy-balanced unequal hierarchical clustering (EULC) algorithm is designed for UWSNs to reduce the energy consumption of communication between clusters. EULC generates clusters of different sizes in the same layer to address the “hot zone” problem. Based on the communication radius, the depth of network deployment is classified into multiple layers. The sensor node calculates the weight regarding the node degree, the distance to the sink node, and the residual energy of the node. The node with the highest weight is selected as the cluster head. Nodes store information about neighboring cluster heads and then calculate the routing function of neighboring cluster heads based on the distance between the residual energy and the node. Moreover, nodes select the next hop cluster head with the minimum routing function value. This algorithm considers the uneven distribution of nodes at each layer but does not consider the node distribution of the whole network and the effect of the coverage redundancy of cluster heads on the network coverage.

In [22], a hierarchical classification-based clustering algorithm (UCUBG) is proposed to select cluster heads according to the density and residual energy of nodes. Then, the cluster head hierarchy is classified based on the location relationship between cluster heads. Finally, nodes select the cluster head with the lowest load into the cluster. This algorithm ignores the influence of cluster head coverage redundancy on network coverage, and only considers the number of neighboring nodes in a certain area when selecting cluster heads, without considering the influence of the distance between the node and its neighboring nodes on the node density.

In [23], the authors implemented a hierarchical adaptive energy efficient cluster routing (HAECR) algorithm. With its hierarchical region division based on node depth in 3D space and cluster head selection based on competition radii and remaining energy, the algorithm is carried out to form clusters of different sizes. In the routing phase, relay nodes are selected from the next layer of cluster heads based on node degree, distance between nodes and remaining energy of nodes. The algorithm effectively mitigates the hotspot problem and further optimizes the uneven transmission load of cluster heads. However, the effect of cluster head coverage redundancy and the influence of a dynamic underwater environment are not considered.

The above-proposed clustering algorithms solve the energy consumption problem to some extent, but few algorithms consider the impact of uneven distribution of sensor nodes on clustering. The EULC and UCUBG algorithms consider the influence of the number of neighbor nodes on clustering but do not consider the problem of the distance from the node to the cluster head, as well as the impact of the coverage redundancy of cluster heads. The HAECR algorithm outperforms UCUBG in terms of balancing network energy consumption but only in static scenarios. This algorithm, combined with the number of neighboring nodes and the distance from the actual dynamic environment, constructs the density function to address the impact of uneven node distribution on cluster splitting and also introduces the coverage redundancy index to handle the impact of easy death of cluster heads on the detection area. It can be effective in prolonging the network lifetime and solving the problem of uneven energy consumption of cluster heads under the uneven distribution of sensor nodes. A qualitative comparison of the clustering protocols is presented in Table 1.

## 3. Underwater Sensor Network Model

**Figure 1:**
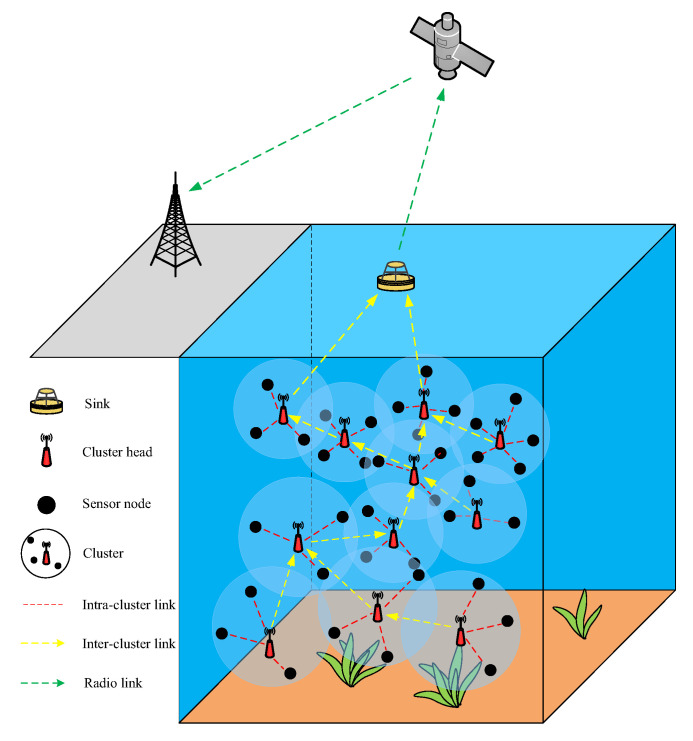
UWSN model.

The network scene consists of N dynamic nodes; each node numbered can be represented as a set $S=\left\{s_{1},s_{2},\ldots ,s_{N}\right\}$, and sink can be represented as $s_{0}$. These nodes are randomly deployed in the 3D scene L×L×L. Through the clustering algorithm, cluster heads are selected, and the topology of different clusters is formed based on the selected cluster heads. The member nodes in the cluster transmit data to the cluster head nodes in a single-hop manner. Then, the cluster head nodes fuse the data sent by all the member nodes in the cluster and use other cluster heads as relay nodes and transmit the data to the sink in a multi-hop manner, as shown in Figure 1. The underwater sensor nodes communicate with other nodes in the water medium using an acoustic modem. The sink nodes are equipped with both acoustic and radio frequency (RF) modems and deployed at the center of the water surface. The acoustic modem receives data from the underwater sensor nodes and the RF modem sends data to the satellite, which in turn sends data to the base station on shore. The underwater sensor nodes are all free to move in the horizontal direction at a speed of 1 m/s. The hypotheses of the network can be described as follows:

- Each node has a unique ID, the transmission range is spherical and is otherwise isomorphic;
- The network operates without considering the sink energy loss;
- Within the communication radius, the nodes can freely adjust the transmission power to save energy consumption;
- The link is symmetric, and the nodes can judge the distance between the two sides based on the received signal intensity.

Acoustic waves are used as a medium by the energy consumption model for data communication in underwater networks [24]. The energy consumption of a node that sends a data packet is

(1) $$E_{t}\left(d\right)=\lambda_{t}lA\left(d\right),$$

where $\lambda_{t}$ is the transmit energy consumption coefficient; l represents the length of the packet; d stands for the distance the packet needs to be transmitted; and $A\left(d\right)$ refers to the energy attenuation of the packet when the underwater transmission distance is *d*, which is denoted as

(2) $$A\left(d\right)=d^{\eta }a^{d},$$

(3) $$a=10^{\frac{a\left(f\right)}{10}},$$

(4) $$a\left(f\right)=0.11\frac{f^{2}}{1+f^{2}}+44\frac{f^{2}}{4100+f^{2}}+2.75\times 10^{-4}f^{2}+0.003,$$

where $a\left(f\right)$ is absorption coefficient, η represents energy dispersion factor, and f stands for carrier frequency.

Below is the energy consumption of the node which receives the packet:

(5) $$E_{r}=\lambda_{r}l.$$

The dynamic nature of the underwater environment leads to different node densities in each network region. The clustering algorithm itself has the problem that its cluster head nodes are significantly different from ordinary nodes in energy consumption. Therefore, the approach to balancing the energy consumption of the network is important for the clustering routing algorithm. Meanwhile, cluster head nodes are prone to death; ensuring the fault tolerance of the cluster head monitoring area is also worthy of attention. Thus, this paper focuses on how to reasonably select cluster heads and design cluster sizes, accounting for the differences between ordinary and cluster head nodes.

The concept of weight is introduced for each node to select a candidate cluster head. A node is chosen as a candidate cluster head when its weight satisfies certain conditions. Below is the condition for the node $s_{n}$ to be a candidate cluster head:

(6) $$F\left(s_{n}\right)=\left\{\begin{array}{c} 1,\;w_{n}^{c}>\mu_{n},\;\mu_{n}\in \left[0,\;1\right],\;n=1,2,\ldots ,N\\ 0 \end{array}\right.$$

where $F\left(s_{n}\right)=1$ means that node $s_{n}$ is chosen as a candidate cluster head; $\mu_{n}$ denotes a random number between 0∼1 generated by the nth node; and $w_{n}^{c}$ represents node weight. The set of candidate cluster heads selected as per the above formula can be defined as $H=\left\{h_{1},h_{2},\ldots ,h_{M}\right\}$, M≤N (M is the number of candidate cluster heads). Any element in the set is written as $h_{m}$.

The size of each cluster is controlled, and nodes that are mutually too close are prevented from becoming cluster heads simultaneously by introducing the contention radius $R_{m}$ of the candidate cluster head and electing the final cluster head. The candidate cluster head becomes the final cluster head directly if no other candidate cluster heads are within its contention radius. It is necessary to compare their remaining energy size if other candidate cluster heads are within the contention radius of the candidate cluster head. The one with the most residual energy succeeds in the election and becomes the final cluster head which is chosen as follows:

(7) $$z_{u}=\left\{\begin{array}{c} h_{m},\;D_{mi}>R_{m},\;i\in \left[m+1,M\right],h_{m}\in B_{u}\\ \underset{h_{i}\in H_{m}}{arg\;max}\left\{EN\left(h_{m}\right),EN\left(h_{i}\right)\right\} \end{array}\right..$$

The set of final cluster heads can be defined as $Z=\left\{z_{1},z_{2},\ldots ,z_{U}\right\}$, U≤M (U is the number of final cluster heads); $D_{mi}$ denotes the distance between candidate heads $h_{m}$ and $h_{i}$; $R_{m}$ represents the contention radius of candidate head $h_{m}$; $B_{u}$ stands for the set of remaining candidate heads after each competition; and $H_{m}$ refers to other I ($I=\left|H_{m}\right|,1\leq I\leq \left|B_{u}\right|-1$) candidate heads within the contention radius of candidate head $h_{m}$.

Each candidate cluster head that has participated in the competition will exit the H-set and not take part in the competition with other ones. Below is the expression of the remaining candidate cluster heads:

(8) $$B_{u}=\left\{\begin{array}{cc} H, & u=1\\ B_{u-1}\backslash A_{u-1}, & u>1 \end{array}\right.$$

where $A_{u}$ denotes the set of elements that exits from set H each time, which can be expressed as

(9) $$A_{u}=\left\{\begin{array}{c} h_{m},D_{mi}>R_{m},\;i\in \left[m+1,M\right]\\ \left\{h_{m}\right\}\cup H_{m} \end{array}\right..$$

Finally, the concept of weight is introduced by considering the relationship between a node and all of its cluster heads within its communication radius. In this way, ordinary nodes can choose the optimal cluster head to join. In addition, ordinary nodes measure the weight value relationship with all cluster heads, select the best cluster head to join and become its member nodes. Below is the condition for an ordinary node to join this cluster head:

(10) $$G_{pq}=\underset{q\in \left[1,U\right]}{arg\;min}(w_{pq}^{h}),p=1,2,\ldots ,\left(N-U\right)$$

where $G_{pq}$ denotes node $s_{p}$ that joins cluster head $z_{q}$; $w_{pq}^{h}$ represents the weight between ordinary node $s_{p}$ and cluster head $z_{q}$.

From the above description, it can be seen that reasonable cluster heads have been selected from the network and appropriate node members for each cluster head have been added. By this means, clusters of different sizes are formed based on the weight relationship between nodes and cluster heads.

## 4. Algorithm Description

NUC-EB, a clustering routing algorithm that is improved, is proposed based on the UCUBG clustering algorithm model proposed in [19]. In this algorithm, the selection of cluster heads by considering the number of adjacent nodes and the remaining energy of nodes is combined with UCUBG. Then, the concepts of node density and coverage redundancy are introduced, which can increase the reasonability of cluster head distribution and ensure the coverage of the network operation process. Additionally, this algorithm also combines the actual dynamic environment to ensure the efficiency and effectiveness of data transmission by dividing the forwarding area and designing the network maintenance mechanism.

### 4.1. Clustering

#### 4.1.1. Choice of Candidate Cluster Leaders

Cluster head selection becomes more reasonable by introducing the concept of node density. The concept of node coverage redundancy is then introduced to improve the fault tolerance of the cluster head monitoring area. A node first broadcasts its residual energy, node ID and location information to its adjacent nodes within the communication radius. The weight of the node will become zero if its residual energy is lower than the average residual energy of all network nodes. Then, it directly drops out of the competition and becomes a member node. Nodes that are qualified for participating in the competition of candidate cluster heads calculate weight size by computing energy, density and coverage redundancy. The weight value of the node $s_{n}$ is expressed as

(11) $$w_{n}^{c}=\left\{\begin{array}{cc} a_{1}\frac{E_{n}^{r}-E_{n}^{av}}{E_{n}^{av}}+b_{1}\frac{\Upsilon_{n}}{\Upsilon_{max}}+c_{1}\frac{\Omega_{n}}{\Omega_{max}}, & E_{n}^{r}\geq E_{n}^{av}\\ 0, & E_{n}^{r}<E_{n}^{av} \end{array}\right.$$

where $E_{n}^{r}$ and $E_{n}^{av}$ denote the remaining and average remaining energy of nodes, respectively; $\Upsilon_{max}$ represents the maximum density of nodes; $\Omega_{max}$ stands for the maximum coverage redundancy of nodes; $a_{1}$, $b_{1}$, $c_{1}$ are adjustment factors, and $a_{1}+b_{1}+c_{1}=1$.

Node density refers to the density of a node, which is up to the number of neighboring nodes within its communication range and the distance from neighboring nodes to that node. The formula for node $s_{n}$ density is expressed as

(12) $$\Upsilon_{n}=\frac{V^{2}}{\sum_{v=1}^{V}D_{nv}},$$

where V is the number of nodes adjacent to the node; $D_{nv}$ represents the distance from the node to its neighboring one $s_{v}$.

Node coverage redundancy is the degree to which the set of nodes adjacent to a node covers its sensing area. The distance to neighboring nodes and the sensing radius can be calculated to obtain the node coverage redundancy. The formula for coverage redundancy is given in the citation [17].

A random number $\mu_{n}$ is defined in the equation according to the method of (6) to determine whether it becomes a candidate cluster head. In this case, the nodes in the sparse region also have the possibility of becoming cluster heads, which avoids the concentration of all cluster heads in relatively dense places at the beginning of the network. From the formula, it can be seen that a node will be more likely to become a candidate cluster head when its weight is larger.

#### 4.1.2. Determination of Cluster Heads

In a multi-hop sensor network, cluster heads near the sink find it necessary to forward data from other more distant ones and thus will consume energy faster. Meanwhile, cluster heads with higher densities will, in turn, consume energy faster than those with relatively lower densities. As a result, it is necessary to close the distance between cluster heads and the sink and reduce the size of denser cluster heads to balance the energy consumption of all cluster heads in the network. In addition, nodes that are very near each other should be stopped from becoming cluster heads. Thus, the competitive radius $R_{m}$ of candidate cluster heads is designed as follows:

(13) $$R_{m}=(1-a_{2}\frac{D_{max}-D_{m0}}{D_{max}-D_{min}}-b_{2}\frac{\Upsilon_{m}}{\Upsilon_{max}})R_{max},$$

where $D_{min}$ and $D_{max}$ denote the minimum and maximum distances from nodes to the sink in the network, respectively; $D_{m0}$ represents the distance between the candidate cluster head $h_{m}$ and the sink; $R_{max}$ stands for the maximum communication radius of nodes; $a_{2}$, $b_{2}$ are adjustment factors, and $a_{2}+b_{2}=1$.

The candidate cluster head broadcasts a message that it has become a candidate cluster head to the nodes in the contention radius. If the candidate cluster head receives no messages from other ones, it means that no other candidate cluster heads are in this contention radius. If the candidate cluster head also receives messages from other nodes to become a candidate cluster head, it means that they are in the same contention radius. The set of final cluster heads can be selected through Equation (7). For the convenience of illustrating the process of the final cluster head campaign, its pseudo-code representation is given below (see Algorithm 1).
**Algorithm 1. Campaign for the final cluster head****Input**: Candidate cluster head set $H\left(m=1,2,\ldots ,M\right)$, final cluster head set $Z\left(u=1,2,\ldots ,U\right)$ and common nodes set $S^{\prime }\left(n=1,2,\ldots ,M-U\right)$1.  **Initializes**: Z=∅, $S^{\prime }=\emptyset$, $H_{m}=\emptyset$, i=m+1;2.  **While** m≤M do3.    **If** $h_{m}\in H$ then4.        **While** i≤M do5.        **If** $D_{mi}>R_{m}$ then6.          i=i+1;7.        **Else If** $D_{mi}\leq R_{m}$ then8.          break;9.        **End While**10.         **If** i=M+1 then11.        $z_{u}=h_{m}$, $H\leftarrow H\backslash h_{m}$;12.         **Else If** i≤M then13.        **While** i≤M do14.             **If** $D_{mi}\leq R_{m}$ then15.            $H_{m}\cup \{h_{i}\}$;16.             i=i+1;17.        **End While**18.        $z_{u}=arg\underset{h_{i}\in H_{m}}{max}\left\{EN\left(h_{m}\right),EN\left(h_{i}\right)\right\}$, $H\leftarrow H\backslash \left(\left\{h_{m}\right\}\cup H_{m}\right)$;19.        $S^{\prime }=\left(\left\{h_{m}\right\}\cup H_{m}\right)\backslash z_{u}$, $H_{m}=\emptyset$;20.         **End If**21.         m=m+1, i=m+1;22.     **Else If** $h_{m}\notin H$ then23.         m=m+1, i=m+1;24.   **End While****Output:** $Z\left(u=1,2,\ldots ,U\right)$, $S^{\prime }\left(n=1,2,\ldots ,M-U\right)$

#### 4.1.3. Common Nodes in Clusters

The node that becomes the final cluster head broadcasts its successful campaign to the entire network. The normal node selects the best cluster head by calculating the generation value to the cluster head within the communication range and notifies the cluster head by sending a message to join. The generation value from the node to the cluster head is designed based on their distance, the distance from the sink to the cluster head, and the residual energy of the cluster head. The weight relationship between node $s_{p}$ and cluster head $z_{q}$ can be expressed as

(14) $$w_{pq}^{h}=a_{3}\frac{D_{pq}}{R_{max}}+b_{3}\frac{D_{q0}}{D_{max}}-c_{3}\frac{E_{q}^{r}}{E_{q}^{o}},$$

where $D_{pq}$ represents the distance between the node and cluster head; $D_{q0}$ stands for the distance between the cluster head and sink; $E_{q}^{o}$ denotes the initial energy of the node; $a_{3}$, $b_{3}$, $c_{3}$ are adjustment factors, and $a_{3}+b_{3}+c_{3}=1$.

**Figure 2:**
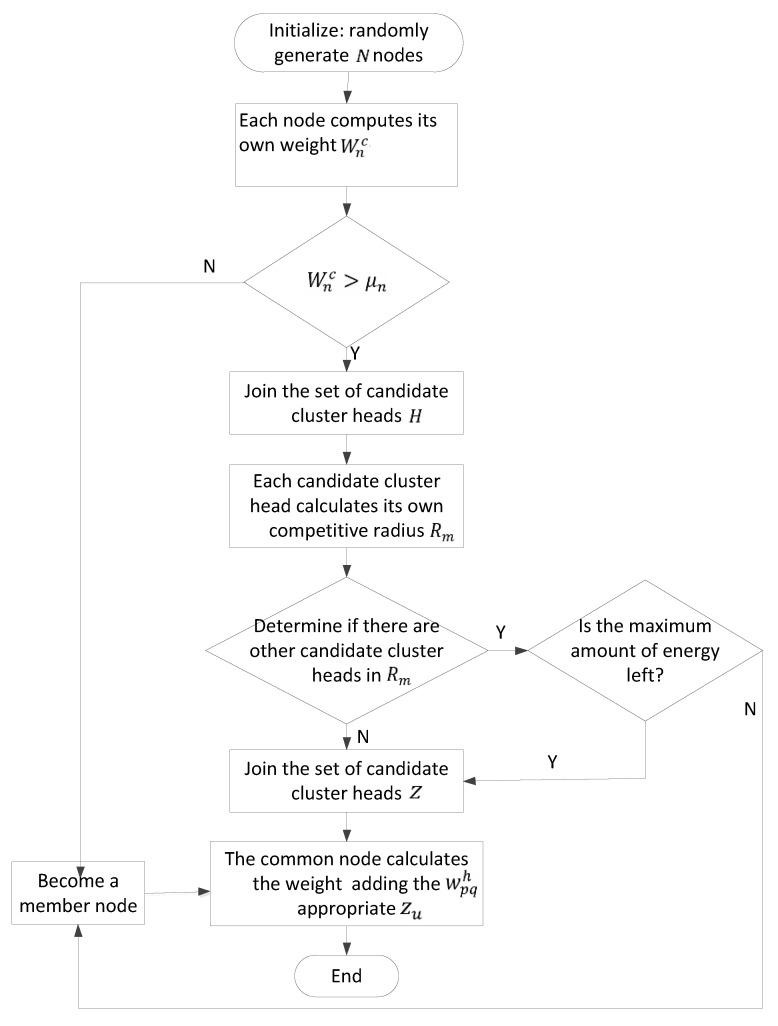
Flow chart of the clustering algorithm.

Through the above calculation, nodes select the cluster head with the minimum corresponding weight into the cluster, as shown in Equation (10), to form a network cluster. The whole process of the clustering algorithm is shown in Figure 2.

### 4.2. Routing

Due to the dynamic nature of underwater nodes, establishing a good complete path directly from the sink is quite inapplicable. Therefore, communication between clusters needs to select the next hop forwarding node in real time using the information within the communication range of nodes. At this point, the selection of the best forwarding node needs to consider the remaining energy of the forwarding node and its distance from the sink.

#### 4.2.1. Division of the Forwarding Area

**Figure 3:**
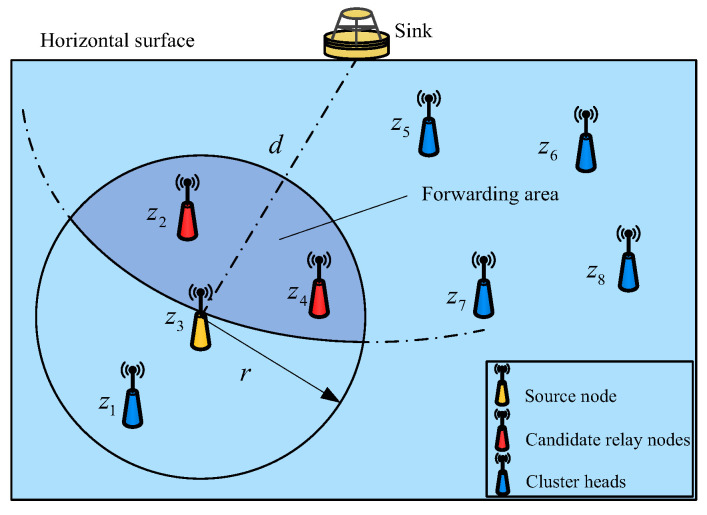
Division of the forwarding area.

To save energy, the routing process employs a multi-hop transmission method. Each time a packet is forwarded, consideration should be given to the distance from the next hop relay node to the sink to make sure that they are closer. Therefore, this algorithm takes the sink as the center and the distance from the sink to the current forwarding node as the radius to simulate a circular arc area. Moreover, the overlapping part of the area covered by this arc and the communication coverage area of the current forwarding node is used as the candidate area of the next hop relay node. For a more visual illustration, a cross-section of a part of the network area is taken as a schematic diagram, as shown in Figure 3. In this figure, $z_{3}$ is the source node; $z_{2}$ and $z_{4}$ are candidate relay nodes in its forwarding area.

Its forwarding area is expressed as follows:

(15) $$\left\{\begin{array}{c} {\left(x-x_{0}\right)}^{2}+(y-y_{0}{)}^{2}+(z-z_{0}{)}^{2}\leq d^{2}\\ {\left(x-x_{1}\right)}^{2}+(y-y_{1}{)}^{2}+(z-z_{1}{)}^{2}\leq r^{2} \end{array}\right.$$

where $\left(x,y,z\right)$ represent the coordinates of candidate next hop nodes in the forwarding area; $\left(x_{0},y_{0},z_{0}\right)$ stand for sink coordinates; $\left(x_{1},y_{1},z_{1}\right)$ denote the coordinates of the current relay node.

#### 4.2.2. Determination of the Best Next Jump

Nodes in the forwarding area reply to the current relay node with its remaining energy and distance from the sink. The relay node is calculated in accordance with the weight formula. The best node is chosen as the next hop relay.

(16) $$W_{k}^{f}=a_{4}\frac{E_{k}^{o}-E_{k}^{r}}{E_{k}^{o}}+b_{4}\frac{D_{k0}}{D_{j0}},$$

where $W_{k}^{f}$ represents the weight of the relay node in the forwarding region; $D_{j0}$ stands for the distance between the current relay node and the sink; $D_{k0}$ denotes the distance between the candidate relay node and sink in the forwarding region; $a_{4}$ and $b_{4}$ are adjustment factors, and $a_{4}+\;b_{4}=1$. It can be seen that the more remaining energy there is, the more likely the node closer to the sink will be the next hop node.

### 4.3. Network Maintenance

UWSNs have a dynamic structure. Nodes move at random with the flow of water. As a result, cluster structure may change anytime. When the distance between a member node and the cluster head is greater than the communication distance, the member node is not capable of transmitting data to the cluster head and thus loses data. Moreover, the cluster head is likely to die early because of its need to forward massive data and consume energy quickly; then, the network will lose a large amount of data. The method of maintaining and updating clusters is designed based on the above problems.

When failing to send data to its cluster head, a member node will monitor packets from the cluster head. Once hearing a packet from the cluster head, it is going to join this cluster based on the information contained in the packet. The cluster head of each cluster computes the mean remaining energy of its cluster. Once its remaining energy is below the mean remaining energy, that cluster reorganizes, elects a new cluster head and updates routing information.

For the whole network, the sink node will broadcast a reorganization message to all nodes every time cycle to prolong the network survival cycle as the cluster head energy of each cluster is consumed too fast. Clusters will be rebuilt by underwater sensor nodes.

## 5. Simulation Results

The effectiveness of this algorithm is verified through simulation experiments. A comparison is made between this algorithm and UCUBG, LEACH and DBR algorithms on the MATLAB platform. In reference [25], the MATLAB software is described in detail. Simulation is conducted in the form of “rounds”, each of which is classified into network cluster formation and data transmission phases, as well as network maintenance nodes. The network data transmission phase involves collecting information from nodes that are within the cluster, transmitting them to the cluster head, and completing the multi-hop forwarding of data between clusters. The network maintenance phase mainly re-clusters problematic clusters. Table 2 shows the parameters of simulation experiments.

### 5.1. Algorithm Stability Analysis

**Figure 4:**
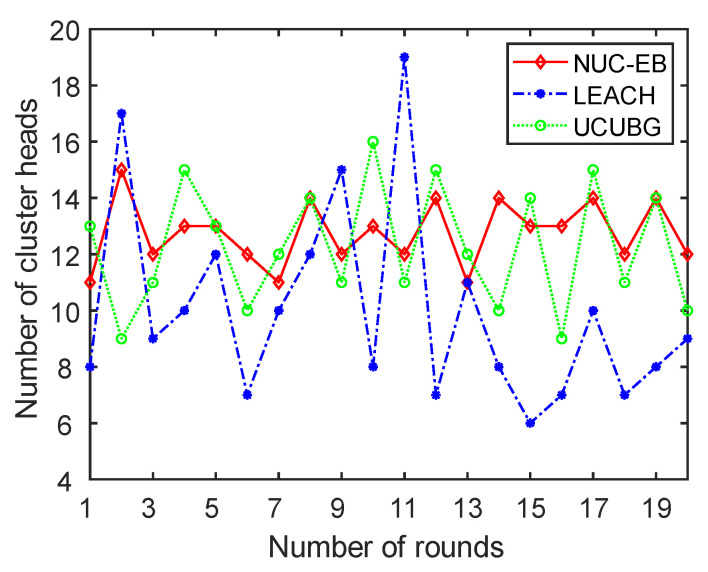
Variation in the number of cluster heads.

In the dynamic selection algorithm of cluster heads, the number of cluster heads selected in every round reflects whether the algorithm is stable. Network stability will be higher when the number of cluster heads is within a smaller variation range. A total of 200 sensor nodes were selected for the experiment, and 20 rounds were randomly selected. Figure 4 shows the variation in the number of cluster heads for the three algorithms.

As shown in Figure 4, the variation ranges of the number of cluster heads of LEACH, UCUBG and the present algorithm are [6, 19], [9, 16] and [11, 15], respectively. Compared with the LEACH algorithm, UCUBG and the present algorithm have better stability. In particular, the stability of the present algorithm is the most obvious. The LEACH algorithm applies a probability function to select cluster heads randomly, which cannot guarantee that the number of cluster heads is stable. UCUBG and the present algorithm have the influence of considering a variety of factors, which makes the choice of cluster head nodes more certain. Compared with the UCUBG algorithm, this algorithm considers the distance between nodes in addition to the number of neighboring nodes when choosing cluster heads, constructs a node density weight function and increments the node coverage redundancy index. In this way, cluster heads in the network are distributed more reasonably and this algorithm has a better stability.

### 5.2. Algorithm Equilibrium Energy Analysis

**Figure 5:**
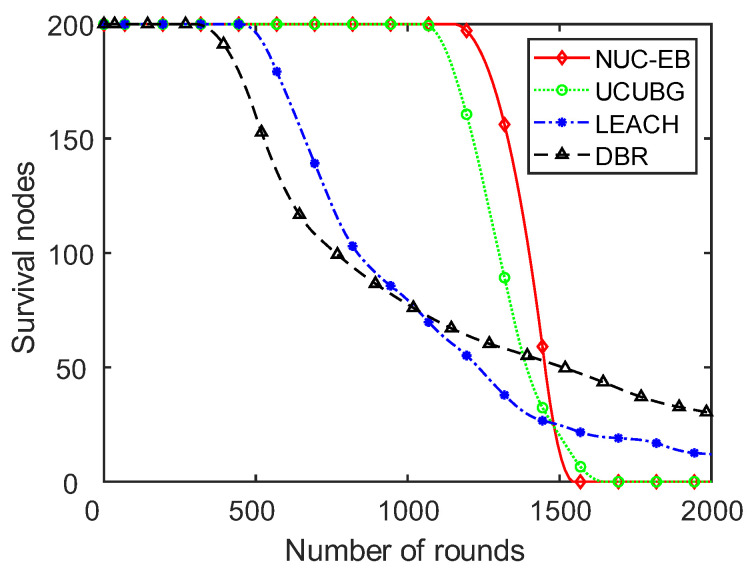
Variation in the number of surviving nodes.

The variation in the number of surviving nodes with the increasing number of rounds in DBR, LEACH, UCUBG and the present algorithm is shown in Figure 4. DBR is a planar structure algorithm and the remaining three are all clustering algorithms. As shown in Figure 5, the DBR algorithm has the earliest node death. A large number of nodes still survived until 2000 rounds. This indicates that the DBR algorithm, a planar structure, has a problem—the poor balancing of network energy consumption, which causes a large difference in node energy consumption and the premature death of some nodes. Among the three clustering algorithms with relatively good balanced energy consumption, this algorithm has the latest round number with the first node death. The death time of the last node is the closest to that of the initial one, which indicates that this algorithm has better balanced energy consumption than the other two clustering algorithms.

### 5.3. Algorithm Coverage Analysis

**Figure 6:**
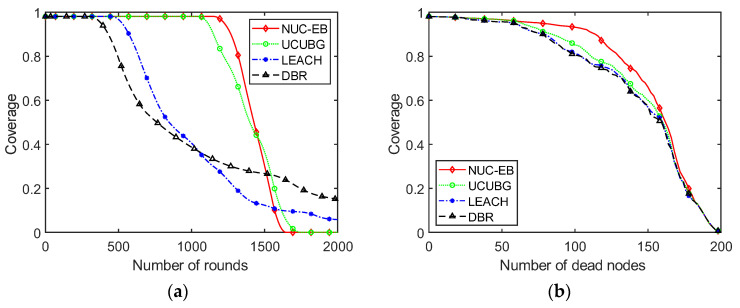
Variation in network coverage: (**a**) with the number of rounds; (**b**) with the number of dead nodes.

The comparison of network coverage with the number of rounds of network operation for DBR, LEACH, UCUBG and the present algorithm is shown in Figure 6a. From it, it can be seen that the network coverage of DBR, LEACH, UCUBG and the present algorithm all decrease with the increasing number of rounds of network operation. Only the LEACH and DBR algorithms have some coverage in the end, which is because these two algorithms run to the end and surviving nodes can still be found. Nevertheless, this algorithm is better than other algorithms in terms of network coverage at the middle stage of network operation. This is mainly due to the slower death rate of nodes in this algorithm and the higher number of surviving nodes in the network. Thus, network coverage is better.

The comparison of network coverage with the number of dead nodes for DBR, LEACH, UCUBG and the present algorithm is shown in Figure 6b. It can be seen from it that the present algorithm has higher network coverage than the other three when the same number of nodes die. This is because the coverage redundancy index is introduced in the selection of cluster heads in this algorithm. Thus, nodes with a high coverage redundancy have a higher likelihood of becoming cluster heads. The network coverage of other algorithms has a greater impact than that of this algorithm in the case of the same number of dead nodes in the network. Therefore, the network coverage of this algorithm is superior to that of the other three for the same number of dead nodes. The UCUBG algorithm has the second-best coverage because it also considers the number of neighboring nodes to choose cluster heads. Nodes with a high number of neighboring nodes have higher coverage redundancy to some extent. Compared with the DBR planar routing protocol, the LEACH algorithm has slightly better coverage. The reason for it is that LEACH has a rotation of cluster head nodes in each round, while the DBR algorithm flooding routing falls into the local optimum.

In addition, network coverage is almost constant when nodes just start to die. This is because the density of network nodes is high, and the coverage redundancy of each node is relatively high in the beginning. When nearly half of the nodes die, the advantage of considering coverage redundancy is more obvious. Meanwhile, the network is sparser, and the impact of coverage redundancy is smaller when fewer nodes remain.

### 5.4. Algorithm Throughput Analysis

**Figure 7:**
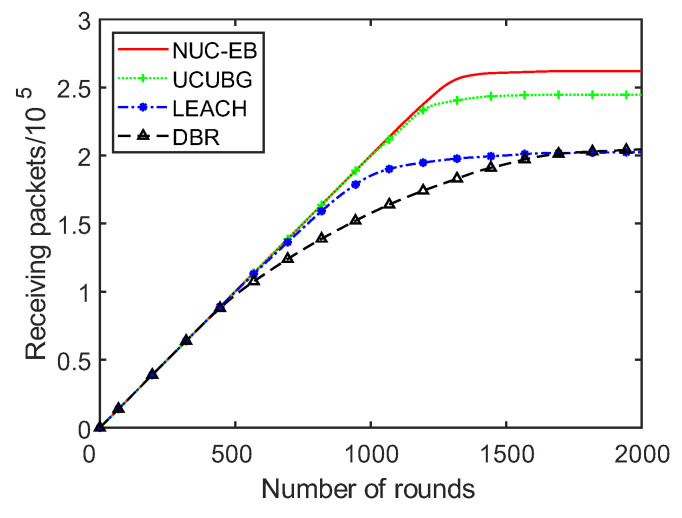
Received packets at the sink.

The number of packets that are received by the sink of DBR, LEACH, UCUBG and this algorithm is shown in Figure 7. It can be seen from it that the number of packets sent to the sink by DBR, LEACH, UCUBG and this algorithm is the same before the death of nodes. However, the number of packets received by the sink of this algorithm is larger than that received by the sink of DBR, LEACH and UCUBG. This is because this algorithm is superior to the other three in the aspect of balanced energy consumption. Nodes in this algorithm start to die later than those in the other three. In addition, this algorithm has a longer network life cycle than the other three.

## 6. Conclusions

This paper proposes an energy-consumption-balanced clustering algorithm. The issue of uneven energy consumption of nodes due to the uneven distribution of nodes in underwater sensor networks is solved. Additionally, coverage redundancy is introduced to select cluster heads to improve the coverage tolerance of the cluster head detection area. The simulation results indicate that the present algorithm has better stability, energy consumption balance and coverage maintenance effect.

## Figures and Tables

**Table 1:** Qualitative comparison of clustering protocols.

Protocols	Node Position	Parameters to Select the Cluster Head	Network	Mobility	Intra-Cluster Routing	Inter-Cluster Routing
LEACH	Random	Random value	2D	Static	Single-hop	Single-hop
DEEC	Random	Residual energy	2D	Static	Single-hop	Single-hop
EEMUC	Layer-based and random	Residual energy, node degree, distance to the sink	2D	Static	Single-hop	Multi-hop
EGRCs	Cube-based grid and random	Residual energy, distance to the sink	3D	Dynamic	Single-hop	Multi-hop
NULCPR	Layer-based and random	Node coverage redundancy	3D	Static	Single-hop	Multi-hop
EULC	Layer-based and random	Residual energy, distance to the sink, node degree	3D	Static	Single-hop	Multi-hop
UCUBG	Random	Residual energy, node degree	3D	Static	Single-hop	Multi-hop
HAECR	Layer-based and random	Residual energy, distance to the sink, node locations	3D	Static	Single-hop	Multi-hop
NUC-EB	Random	Residual energy, node density, node coverage redundancy	3D	Dynamic	Single-hop	Multi-hop

**Table 2:** Parameters of simulation experiments.

Parameters	Value
Network area/m^3^	100 × 100 × 100
Maximum communication radius $R_{max}/m$	50
Perception radius $R_{g}/m$	20
Initial energy $E_{0}/J$	2000
Signal frequency f/kHZ	10
Sensor nodes	200
Launch energy factor $\lambda_{t}$	0.001
Receiving energy factor $\lambda_{r}$	0.001
Packet length l/bit	2000
Nearest distance $d_{min}/m$	0
Longest distance $d_{max}/m$	125

## Data Availability

Not applicable.
